# Acute Cocaine Intoxication Leading to Multisystem Dysfunction: A Case Report

**DOI:** 10.7759/cureus.72128

**Published:** 2024-10-22

**Authors:** Mateus Rodrigues Alessi, Thiago Malheiros Ribas, Victor Setti Campelo, Sivan Mauer

**Affiliations:** 1 School of Medicine, Universidade Positivo, Curitiba, BRA; 2 Internal Medicine, Faculdade Evangélica Mackenzie do Paraná, Curitiba, BRA; 3 Psychiatry, Faculdade Evangélica Mackenzie do Paraná, Curitiba, BRA

**Keywords:** cocaine-related disorders, disseminated intravascular coagulation, drug overdose, malignant hyperthermia, recreational drug use, rhabdomyolysis

## Abstract

Cocaine intoxication presents a significant public health concern globally, with its use widespread in various regions. This case report outlines the challenging management of a 30-year-old male who presented with acute cocaine intoxication, leading to multiple organ failure and eventual death. The patient exhibited classic symptoms of cocaine intoxication preceding a fall, resulting in severe injuries. Despite aggressive resuscitative measures, the patient deteriorated rapidly, developing rhabdomyolysis, disseminated intravascular coagulation, acute kidney injury, and acute myocardial infarction. Differential diagnoses were considered, ruling out other possible causes. The patient’s condition continued to worsen despite treatment, ultimately culminating in cardiac arrest and demise. This case underscores the complexity of managing acute cocaine intoxication and assessing many differential diagnoses, highlighting the importance of early recognition and aggressive intervention to mitigate adverse outcomes. In patients with altered mental status and hemodynamic instability, particularly in areas where drug use is common, cocaine intoxication should be considered as a possible cause.

## Introduction

Cocaine is a semi-synthetic drug manufactured from the leaves of the *Erythtoxylon coca* plant along with various chemical solvents. Brazil ranks second globally in cocaine consumption, consuming 18% of the world’s production, second only to the United States, where nearly 30,000 people die from cocaine overdose each year [1]. Its domestic consumption surpasses the entire of Asia and South America, excluding Brazil. The illicit drug also ranks second as the most consumed drug in the country [1]. Recent research indicates that 3.1% to 3.9% of the population has experimented with the substance in some form [2]. Its effects appear within three to five seconds when inhaled, 10 to 60 seconds when injected intravenously, and four to five minutes when snorted, but it presents prolonged effects due to local vasoconstriction, lasting up to 90 minutes [3]. Its main symptoms stem from the activation of alpha-1 adrenergic receptors and inhibition of dopamine reuptake as the primary mechanism, along with the reuptake inhibition of serotonin and norepinephrine initially leading to sympathetic nervous system activation associated with tachycardia, hypertension, paranoia, psychosis, mania, and delirium [3]. However, it can progress to multiple organ dysfunction due to ischemia and free radical formation, a condition of poor prognosis [4].

Here, we present a case of difficult management resulting from acute cocaine use, leading to multiple system failure and culminating in the patient’s death.

## Case presentation

A 30-year-old Caucasian male patient arrived at the hospital at 12:37 PM by ambulance after an accidental fall from a height of 2 m during work. He was intubated, with a blood pressure of 60/40 mmHg, heart rate of 142 beats/minute, oxygen saturation of 95%, respiratory rate of 32 breaths/minute, temperature of 41.2°C (106.16°F), and capillary refill time >6 seconds. He was sedated, dehydrated, and hypochromic, with abrasions on the upper and lower limbs, along with ecchymosis. His wife had accompanied him and reported that he had exhibited psychomotor agitation, diaphoresis, and hypertensive episodes before the fall. She reported that the patient had stopped using cocaine for the past three months, but had relapsed, using high doses in the last few weeks, including snorting cocaine within the past few hours. The wife reported that he was only using cocaine and denied the use of any other illicit drugs. During physical examination, no signs of nasal septal perforation were observed.

**Figure 1:**
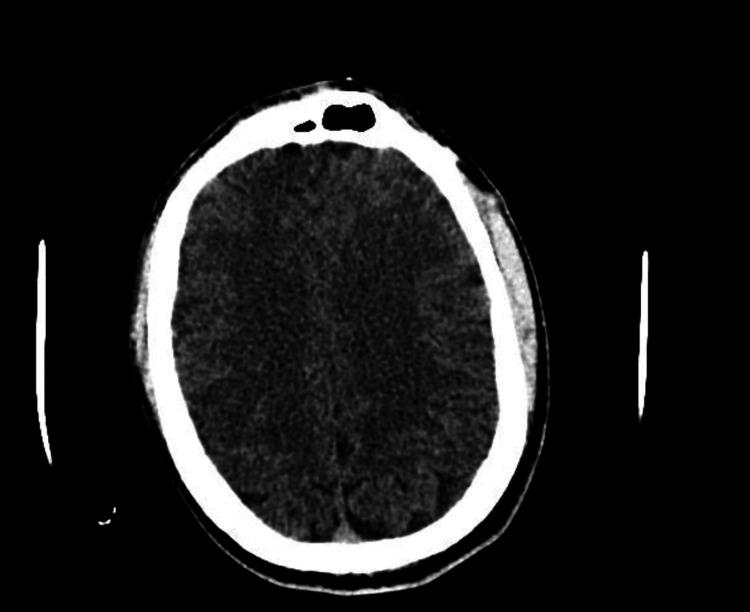
Head CT scan showing no abnormalities. CT scan of the skull and brain was performed using a multidetector helical technique. No contrast medium was administered.

After admission and initial stabilization, laboratory and imaging tests were performed. A CT scan of the head showed no evidence of intracranial expansive processes, acute intraparenchymal hemorrhages, midline structure deviation, or signs of displaced skull fractures (Figure 1).

**Figure 2:**
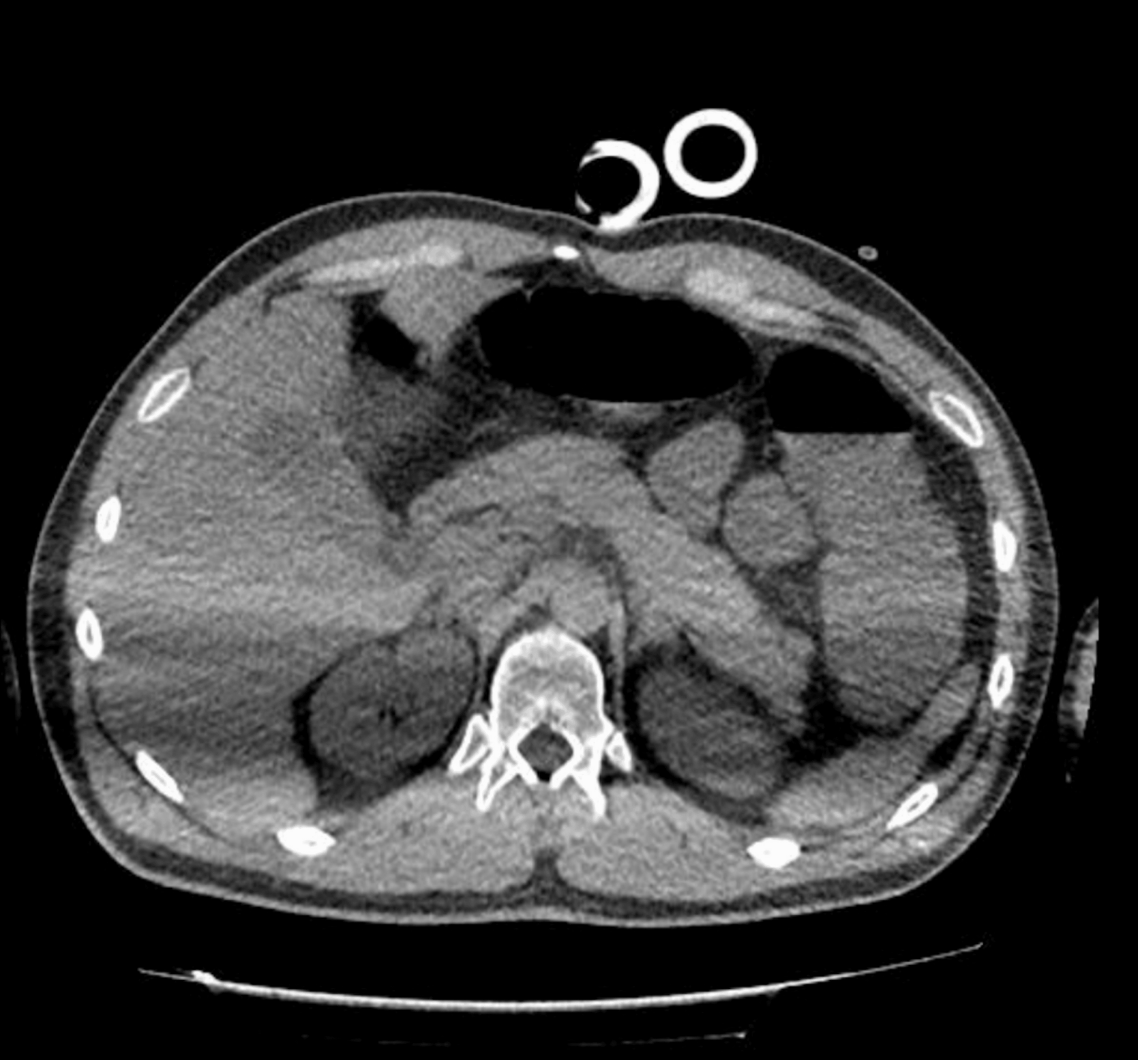
Abdominal CT scan showing no abnormalities. CT scan of the upper abdomen and pelvis was performed using a multidetector helical technique. Images were obtained before and after intravenous administration of the contrast medium.

**Figure 3:**
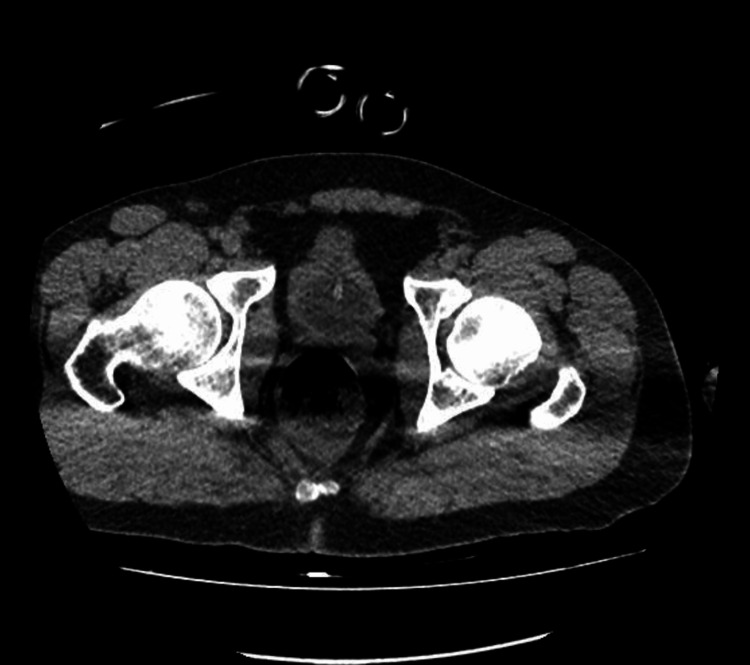
Pelvic CT scan showing no abnormalities. CT scan of the upper abdomen and pelvis was performed using a multidetector helical technique. Images were obtained before and after intravenous administration of the contrast medium.

**Figure 4:**
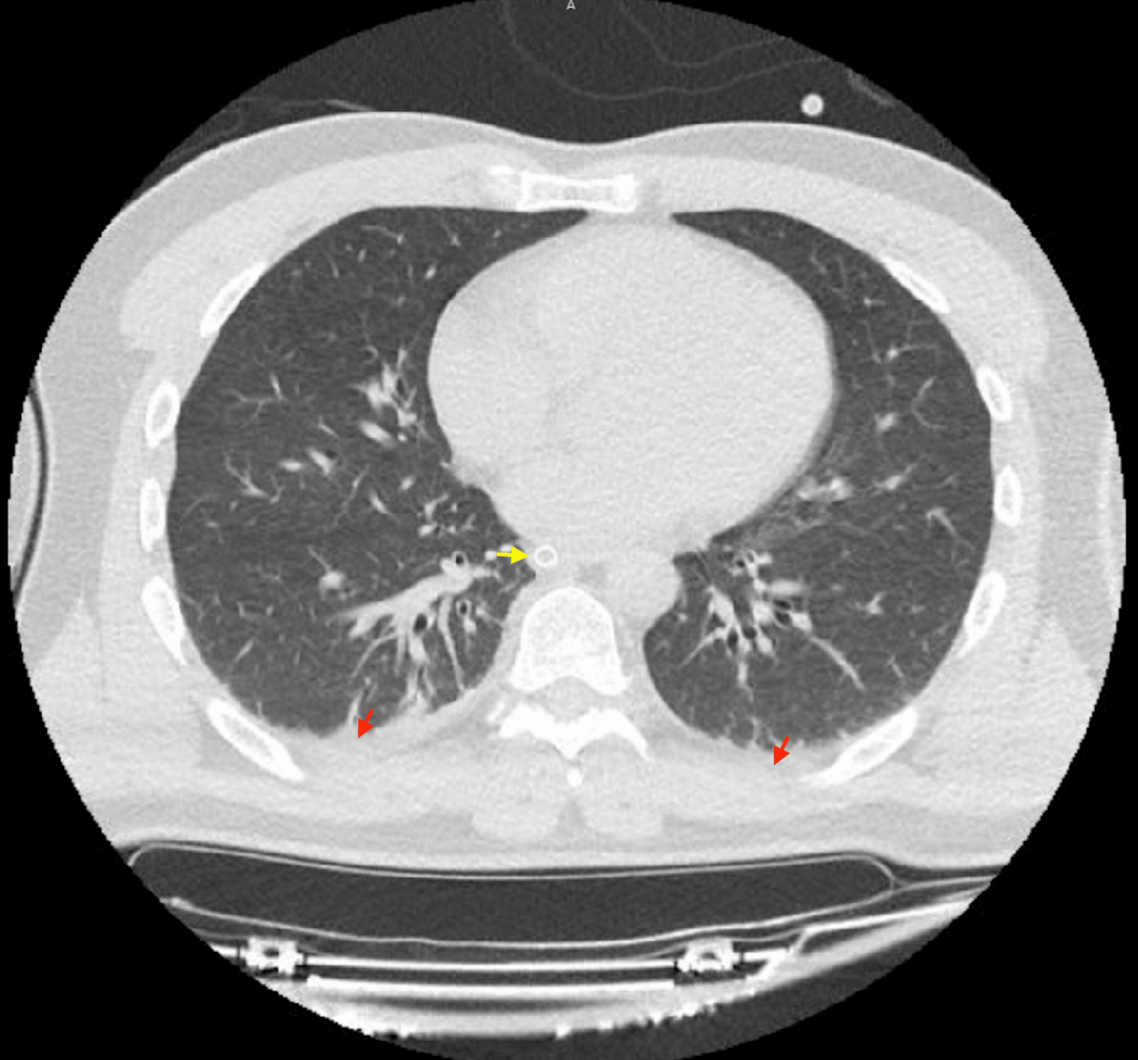
Chest CT scan. Volumetric acquisition using multi-detector equipment with thin slices and high-resolution images. No contrast medium was administered. The red arrow shows a small pleural effusion, and the yellow one indicates the orotracheal tube. Apart from these signs and a right clavicle fracture sequela (not visible in this figure), the official report indicated no abnormalities.

His CT scans of the abdomen and pelvis were also normal, without evidence of free fluid, pneumoperitoneum, or organized collections in the abdominal cavity. The chest CT showed laminar pleural effusion on the right, as well as a sequela of a previous right clavicle fracture. There were no signs of pneumothorax or aortic dissection (Figures 2-4). His initial laboratory tests indicated leukocytosis, acute kidney injury, uncompensated metabolic acidosis, coagulopathy, hypoglycemia, and elevated transaminases and lipase (Table 1).

**Table 1:** Evolution of the laboratory tests. CBC = complete blood count; Na = sodium; K = potassium; Ca = calcium; Mg = magnesium; Cl = chlorine; Cr = creatinine; Ur = urea; ABG = arterial blood gas; HCO_3^-^_ = bicarbonate; BE = base excess; CPK = creatine phosphokinase; ALT = alanine aminotransferase; AST = aspartate aminotransferase

Examinations	12/11/23, 01:37 PM	12/11/23, 09:57 PM	13/11/23, 10:53 AM	Laboratory test reference values
CBC				
Hemoglobin (g/dL)	16.4	-	15.3	13.5–17.8
Leukocytes (/µL)	32,100	-	31,000	3,600–11,000
Platelets (/µL)	165,000	-	67,000	150,000–450,000
Electrolytes				
Na (mEq/L)	145	-	150	136–145
K (mEq/L)	4.2	-	4.61	3.5–5.1
Ca (mEq/L)	8.93	-	-	8.8–10.6
Ionized calcium (mEq/L)	-	-	0.83	1.16–1.31
Mg (mEq/L)	2.57	-	-	1.8–2.6
Cl (mEq/L)	-	-	117	96–106
Renal function				
Cr (mg/dL)	3.35	4.58	6.11	0.67–1.17
Ur (mg/dL)	63	88	113	17–43
Coagulogram				
INR	2.01	-	2.01	1.0–1.3
PTT (seconds)	-	-	61.7	30.5–36.5
Fibrinogen (mg/dL)	-	-	32.8	200–400
ABG				
pH	7.21	-	6.91	7.350–7.450
PCO_2 _(mmHg)	36	-	44	32–45
HCO_3_^- ^(mEq/L)	14.4	-	8.8	20–26
BE	-13.5	-	-23.9	-2–+2
SaO_2_	95%	0	92%	95–100%
Others				
Lactate (mmol/L)	2.2	4.4	11.21	0.5–2.2
CPK (µg/L)	-	7,753	145,120	<171
Glucose (mg/dL)	43	-	43	>70
Troponin (ng/mL)	-	-	>24,998	<0.012
Lipase (U/L)	169	-	-	<67
ALT (U/L)	52	-	-	<50
AST (U/L)	53	-	-	<40

The patient was transferred to the semi-intensive care unit (semi-ICU) where he underwent continuous hemodynamic monitoring and mechanical ventilation. Fresh plasma, fluid replacement, norepinephrine plus vasopressin, discontinuation of atracurium (which had been used in the ambulance), hydration with lactated Ringer’s solution, hypertonic glucose, and a dantrolene bolus for possible malignant hyperthermia were prescribed. After more than eight hours of hospitalization, the patient experienced worsening renal function (creatinine: 4.58 mg/dL; urea: 88 mg/dL) and increased lactate (4.4 mmol/L). On that day, creatine phosphokinase (CPK) was requested, showing a value of 7,753 U/L, indicative of rhabdomyolysis.

On the morning of the second day (at 10:53 AM), laboratory tests indicated the progression of the condition, evidenced by worsening renal function, disseminated intravascular coagulation, severe uncompensated metabolic acidosis, and a significant increase in CPK (145,120 U/L) and troponin (>24,998 U/L). These demonstrated a severe multiple organ failure that was not being stabilized or delayed, even with treatment. Later that afternoon (at 4:04 PM), the patient presented with monomorphic ventricular tachycardia without a pulse, and therefore, electrical cardioversion and cardiopulmonary resuscitation were performed. After nine minutes and 18 seconds of cardiovascular resuscitation, the patient returned to spontaneous circulation. Unfortunately, at 10:47 PM on the same day, the patient passed away due to a new cardiac arrest unresponsive to resuscitation measures.

No urine drug tests were performed, as they are not available in public hospitals for emergencies in Brazil. Additionally, his blood alcohol level was not measured because this test is also unavailable for emergency care.

## Discussion

The main symptoms of acute cocaine intoxication result from the catecholaminergic discharge produced by the drug, activating the sympathetic nervous system, causing mainly hypertension, diaphoresis, tachycardia, and agitation [5]. However, its clinical presentation can often overlap with other diseases. Some key signs may draw attention to acute intoxication, instead of differential diagnoses, such as mydriasis, lesions on the nasal septum, patient in a risky situation, and neurological symptoms (psychosis, hallucinations, mental confusion) in a patient demonstrating hypertension, sweating, and psychomotor agitation (Table 2). The main differential diagnoses are discussed in Table 3.

**Table 2:** Main signs and symptoms of cocaine intoxication. The table shows the main signs and symptoms that doctors should observe when suspecting acute cocaine intoxication [5].

Mild-to-moderate intoxication	Severe intoxication
Agitation	Severe agitation or seizure
Hallucinations	Arrhythmias
Anxiety	Coagulopathy
Diaphoresis	Cardiovascular collapse
Chest pain	Acute renal failure
Mydriasis	Liver injury
Nausea and vomiting	Rhabdomyolysis
Palpitation	Reduced cardiac function
Tachycardia	Tachycardia
Tachypnea	Tachypnea
Hypertension	Hypertension
Hyperthermia	Hyperthermia

**Table 3:** Differential diagnoses of acute cocaine intoxication. The table indicates the main differential diagnoses that might look similar to acute cocaine intoxication [6-14].

Differential diagnoses
Malignant hyperthermia
Serotonin syndrome
Malignant neuroleptic syndrome
Amphetamine intoxication
Alcohol and sedative-hypnotic withdrawal syndromes
Synthetic cannabinoid intoxication
Pheochromocytoma

Pheochromocytoma is a tumor related to the overproduction of catecholamines, which are hormones that can cause significant increases in blood pressure and heart rate. Peak pressure episodes occur intermittently and are related to hyperglycemia. This condition is not related to rhabdomyolysis, mental confusion, or renal failure, as described in the case [6].

Malignant hyperthermia is characterized by a significant elevation in body temperature, with a risk of death, and may be associated with tachypnea, tachycardia, nausea, vomiting, and mainly muscular rigidity [7-9]. Malignant hyperthermia is usually associated with the administration of anesthetic medications, such as muscle relaxants, but it is more associated with succinylcholine than atracurium [10]. It is worth mentioning that the patient did not demonstrate muscular rigidity and did not improve after the initiation of dantrolene and the suspension of muscle relaxant. In cases where malignant hyperthermia is identified and promptly treated, fewer than 5% of patients ultimately die. All these factors do not support the diagnosis mentioned above.

Intense amphetamine intoxication can present with mental confusion, erratic speech, headache, psychomotor agitation, and extreme anger, with threats or demonstrations of aggressive behavior, symptoms very similar to those of cocaine intoxication [11]. However, psychosis and hallucination tend to last more than 48 hours. In this case, there was no report of this substance use by the patient.

Cannabinoid intoxication can present with cardiovascular and neurological effects, such as tachycardia, myocardial infarction, seizures, vomiting, arterial hypertension, increase in appetite, and conjunctival injection [12]. These latter symptoms were not present in the patient, and there was also no report of use by the patient.

Alcohol withdrawal is divided into phases. In the mild phase, tremors, tachycardia, arterial hypertension, and agitation occur within 48 hours. The alcoholic hallucination phase is represented by the symptoms mentioned, along with hallucination in the absence of delirium. Finally, delirium tremens is characterized by intense hallucination and autonomic dysfunction, followed by delirium and seizures [13]. Withdrawal syndrome does not involve rhabdomyolysis or renal failure, usually leading to death from seizures or cardiac arrest. As reported by the patient’s companion, he had ingested alcohol on the same day, drinking 800 mL (approximately 27 ounces) of distillate.

Malignant neuroleptic syndrome clinically presents with mental confusion, agitation, coma, muscular rigidity, high temperature (often above 40°C or 104°F), tachycardia, tachypnea, and high blood pressure [14]. In addition, it can lead to acute renal injury due to myoglobin. Nevertheless, it is related to the use of antipsychotics. The case presented many similarities with the malignant neuroleptic syndrome; however, there was no report of antipsychotic use by the patient at any time, and he did not present muscular rigidity, a classic sign of the condition.

Serotonin syndrome occurs due to increased stimulation of serotonin receptors in the brain, usually caused by drug interaction. The clinical presentation of the syndrome is severe, with symptoms such as anxiety, agitation, restlessness, startles, delirium with confusion, tremors or muscle spasms, hyperreflexia, tachycardia, hypertension, high body temperature, sweating, chills, vomiting, and diarrhea [15]. Although the condition is very similar to this patient, he did not show hyperreflexia or come into contact with drugs that could have caused the condition.

Currently, acute cocaine intoxication is divided into three stages, starting with milder and classic symptoms of sympathetic activation and finally presenting with coma, myocardial dysfunction, respiratory failure, and death.

The main protocols in the evaluation of a patient suspected of intoxication involve ABCDE management and patient stabilization. Recommended tests include renal and hepatic function, complete blood count, creatine kinase, troponin, electrocardiogram (ECG), and partial urine with toxicological testing [16]. Depending on each patient’s condition, more tests may be included, such as a chest X-ray in cases of inhalation suspicion, or a CT scan of the head in cases of suspected stroke. As the diagnosis is only conclusively made with toxicological testing, which is not widely available in Brazilian public hospitals, it is extremely important for the medical team to contact family members or acquaintances to understand each patient’s context.

The treatment begins with patient sedation using both benzodiazepines and antipsychotics [17]. As measures for hyperthermia, external cooling is the most indicated, with nebulization using warm water cooled by convection from a fan being the primary choice, but other measures can be used [17]. If blood pressure does not decrease with benzodiazepines, sublingual nitroglycerin may be used. In more extreme cases, nitroglycerin, sodium nitroprusside, phentolamine, or labetalol may be used [18]. Despite labetalol presenting beta-2 blockade, it has been shown to be safe, not causing the controversial reflex tachycardia, supposedly occurring when using beta-blockers for acute cocaine intoxication [18].

Although most cases demonstrate complete recovery in fewer than 24 hours, some intoxications involve dysfunction of multiple organs, as in the presented case. Its main cause is related to local ischemia generated by cocaine [4]. The patient presented acute renal failure due to ischemia in the arterioles, decreasing blood flow, and due to acute tubular necrosis, generated by myoglobin pigment released after rhabdomyolysis [19]. This, which is also generated by tissue ischemia, causes muscular destruction and the release of toxic substances. Disseminated intravascular coagulation is a situation where there is consumption of the structures responsible for hemostasis with thrombus formation at the same time, and is present in situations of severe imbalance of the functioning of the human body systems. Hepatic injury is still not well understood, but it is speculated to be related to ischemia and oxidation of hepatocytes [4]. Finally, the neurological symptoms stem from vasospasm, oxidative stress, accumulation of free radicals, enzymatic blockade, and inhibition of serotonin, noradrenaline, and dopamine reuptake [3]. It would still be possible to speculate on a possible acute myocardial infarction, given the increase in troponin and the occurrence of monomorphic ventricular tachycardia; however, only one troponin collection was performed, and the ECG did not show ST-segment elevation [5]. It is already known that various situations can cause an increase in troponin, such as acute renal injury and rhabdomyolysis, both present in the presented case [20].

It is important to emphasize that the clinical presentation of acute cocaine intoxication can vary greatly from individual to individual, mainly due to the quantity and form of use.

## Conclusions

The presentation of acute cocaine intoxication can be similar to multiple diagnoses, requiring a good anamnesis, physical examination, and laboratory examination for its identification, which should be followed by early treatment. Even though its presentation can vary significantly, all cases require a medical examination, as complications and death are not uncommon.
